# Evaluation of fecal microbiota transplantation in Parkinson's disease patients with constipation

**DOI:** 10.1186/s12934-021-01589-0

**Published:** 2021-05-13

**Authors:** Xiao-yi Kuai, Xiao-han Yao, Li-juan Xu, Yu-qing Zhou, Li-ping Zhang, Yi Liu, Shao-fang Pei, Chun-li Zhou

**Affiliations:** 1 Department of Gastroenterology, The Affiliated Suzhou Hospital of Nanjing Medical University, No. 242, Guangji Road, Suzhou, 215008 Jiangsu China; 2 Department of Neurology, The Affiliated Suzhou Hospital of Nanjing Medical University, No. 242, Guangji Road, Suzhou, 215008 Jiangsu China

**Keywords:** Parkinson’s disease, FMT, Gut microbiota, 16s rDNA sequencing, Constipation

## Abstract

Parkinson’s disease (PD) is a neurodegenerative disorder and 70–80% of PD patients suffer from gastrointestinal dysfunction such as constipation. We aimed to assess the efficacy and safety of fecal microbiota transplantation (FMT) for treating PD related to gastrointestinal dysfunction. We conducted a prospective, single- study. Eleven patients with PD received FMT. Fecal samples were collected before and after FMT and subjected to 16S ribosomal DNA (rDNA) gene sequencing. Hoehn-Yahr (H-Y) grade, Unified Parkinson's Disease Rating Scale (UPDRS) score, and the Non-Motion Symptom Questionnaire (NMSS) were used to assess improvements in motor and non-motor symptoms. PAC-QOL score and Wexner constipation score were used to assess the patient's constipation symptoms. All patients were tested by the small intestine breath hydrogen test, performed before and after FMT. Community richness (chao) and microbial structure in before-FMT PD patients were significantly different from the after-FMT. We observed an increased abundance of *Blautia* and *Prevotella* in PD patients after FMT, while the abundance of *Bacteroidetes* decreased dramatically. After FMT, the H-Y grade, UPDRS, and NMSS of PD patients decreased significantly. Through the lactulose H2 breath test, the intestinal bacterial overgrowth (SIBO) in PD patients returned to normal. The PAC-QOL score and Wexner constipation score in after-FMT patients decreased significantly. Our study profiles specific characteristics and microbial dysbiosis in the gut of PD patients. FMT might be a therapeutic potential for reconstructing the gut microbiota of PD patients and improving their motor and non-motor symptoms.

## Introduction

Parkinson’s disease (PD) is a multifocal, progressive, chronic, neurodegenerative disorder that affects millions of patients worldwide. It is characterized by various motor symptoms including tremors, rigidity, bradykinesia (often akinesia), and postural abnormalities (characterized by a shuffling gait), and always accompanied by gastrointestinal dysfunction such as constipation [1]. Gastrointestinal symptoms in the entericnervous system (ENS) occur during the early stages of PD and was therefore once considered as the origin of the pathological process underlying PD, in contrast to it being a possible PD symptom instead [2].

Based on one study we can state that 70–80% people suffering with PD will have constipation [3]. Previous studies have demonstrated constipation and infrequent bowel movements to be direct risk factors of PD [4]. Since constipation severely affects the PD patient’s overall quality of life [5], effective clinical treatment options are necessary.

Fecal microbial transplantation (FMT) consists of transplanting gut microbiota of healthy people into the patients’ intestines and is currently is an effective treatment for *Clostridium difficile* infection, Crohn’s disease, constipation, as well as certain neurological diseases [6]. Several case reports, retrospective case series, and randomized, controlled trials have demonstrated the benefit of FMT in patients with functional bowel disorders, including constipation [7]. Hongliang et al. [8] demonstrated that FMT was more effective than conventional treatment (education, behavioral modification, oral laxatives, probiotics, and rescue with macrogol) in patients with slow transit constipation (STC).

FMT is a recognized therapeutic option for the functional gastrointestinal disease through gut microbiota reconstruction [9]. Since 2011, Ananthaswamy et al. [10] reported a case report of the symptomatic treatment of Parkinson’s disease through FMT.

Further research demonstrated FMT’s possible important role in reducing disease progression and improving the psychomotor and neurological symptoms in PD patients.

Based on these reports, we speculate FMT to be a potential therapeutic option for improving motor and non-motor symptoms in PD patients with constipation. Therefore, The purpose of the present study was to evaluate the effectiveness and safety of a single fresh FMT for PD patients with constipation.

## Materials and methods

### Learning design and patient qualifications

This prospective study included 11 PD patients with constipation. The protocol was approved by the institutional review committee of The Affiliated Suzhou Hospital of Nanjing Medical University (ChiCTR2000040891). All patients were informed of possible adverse reactions, provided written informed consent, and voluntarily accepted the FMT. Patients were excluded if they had severe immunodeficiency, obvious liver and kidney dysfunction, could not provide informed consent, or were accompanied by *C. difficile* infections or other intestinal pathogens. During the study, other drug treatments were allowed. All the patients basically followed the traditional Chinese Food structure (containing mainly grains and vegetables, small amounts of meat) before and after the FMT treatment.

### Clinical improvement assessment

All PD patients were evaluated by the Hoehn-Yahr (H-Y) grade and Unified Parkinson's Disease Rating Scale (UPDRS) score [11]. Non-motor symptoms were assessed using the Non-motor symptom questionnaire (NMSS) [12]. PAC-QOL score [13] and Wexner constipation score [14] were used to assess the patient's constipation symptoms, and further compare the improvement in the patient's motor symptoms and non-motor symptoms, before and after FMT. All these before mentioned laboratory indicators and scale scores were used to compare the patients' improvement in motor symptoms and non-motor symptoms, before and after FMT.

### FMT procedure and efficacy-safety assessment

Frozen fecal microbiota was obtained from the China fmtBank (Nanjing, China). Around 40 to 50 ml of frozen fecal microbiota was suspended in 200 ml of warm normal saline, fresh every time, and transplanted into the intestine, within 2–4 min of the suspension, through a nasoduodenal tube.

Efficacy-safety Assessment: Patients with adverse events during the treatment or the observation period within 12 weeks of FMT, were instructed to record their discomforts through a daily log and report to the investigators immediately; adverse events including abdominal pain, fever, vomiting, flatulence, nausea, and other gastrointestinal symptoms.

### Fecal sample collection and microbial community analysis

Fecal samples from all of the 11 PD patients before FMT and 4, 8, 12 weeks after FMT, and that of healthy controls (HCs) were collected and stored at − 80 °C until usage. The 16s rDNA sequencing was used for the microbiota analysis. The phylum- and family-level analyses were used to assess the composition of the fecal bacteria. In addition, the Shannon diversity index and chao1 index were used to assess the microbiota diversity.

### The Lactulose H_2_ Breath Test (LHBT) procedure and small intestine bacterial overgrowth (SIBO) diagnosis

Patients were instructed to consume a low carbohydrate diet on the day before **LHBT**, to fast overnight, and avoid ingestion of anything but water for 12 h before the test. A baseline breath sample was obtained, followed by ingestion of 10 g lactulose with up to 100 ml of water. Breath samples were then collected at 15 min intervals for 180 min.

The expiratory breath samples were taken for alveolar gas sampling using an alveolar gas collection system (AGC-3000, Laboratory for expiration biochemistry nourishment metabolism Co., Ltd, Nara, Japan). Samples were analyzed for hydrogen and methane and were reported in parts per million (ppm) by a breath gas analyzer (BGA-1000D, Laboratory for expiration biochemistry nourishment metabolism Co., Ltd, Nara, Japan). Measurements were plotted graphically. The breath test was considered positive if there was an increase in the level of hydrogen gas, > 20 ppm above baseline at any point during the first 180 min.

### Statistical analysis

Data were analyzed by SPSS version 22. Comparison between the groups was conducted using the Student’s t-test. P < 0.05 was considered to be statistically significant. Alpha diversity included observed richness, observed species, and the Shannon and Chao1 indexes using the R package vegan. The significance of differences in measured alpha-diversity metrics across samples was tested using a nonparametric Kruskal–Wallis rank sum test and the Benjamini–Hochberg correction. The taxon abundance was measured and plotted using ggplot2. LEfSe analysis was performed to identify taxa with differential abundance in the different groups. LEfSe is an algorithm for high-dimensional biomarker discovery and explanation that identifies genomic features that characterize the differences between two or more biological conditions. Moreover, indicator analysis based on genera was conducted using the R package. Indicator taxon analysis was a way to calculate the probability that any taxon was found in different groups. The heat map of the Spearman’s rank correlation coefficient was used to describe the specific correlation between clinical indices and selected OTUs.

## Results

### Patients’ characteristics

A total of 11 PD patients with constipation symptoms took part in our study. None of them had abdominal pain, diarrhea, fever, or other adverse reactions. Baseline characteristics, including the Hoehn-Yahr (H-Y) Grade, Unified Parkinson’s Disease Rating Scale (UPDRS) II Score, non-motor symptom questionnaire (NMSS), PAC-QOL score, Wexner constipation score, body mass index (BMI) (mm/kg^2^),homocysteine (HCY), albumen (Alb), and uric acid (UA),were obtained from the 11 PD patients before FMT, and 6 and 12 weeks after FMT. The median disease duration in PD patients was 7.18 ± 3.25 years (range: 1–12 years). Detailed clinical characteristics are presented in Table 1.

**Table 1:** Included patient characteristics at baseline

Patient	Sex (F/M)	Age (years)	Disease duration (years)	BMI
1	F	62	9	23.19
2	M	70	8	20.39
3	M	73	10	23.66
4	F	84	12	19.56
5	F	58	8	22.4
6	M	69	6	20.78
7	F	40	1	22.72
8	M	41	2	20.2
9	M	69	7	22.23
10	M	61	7	25.39
11	M	60	9	21.56
Total (Mean ± SD)	NA	62.45 ± 13.08	7.18 ± 3.25	22.01 ± 1.73

### Basal and six-week assessment

Basal median H-Y Grade was 2.27 (range 1–3), which was higher than patients receiving FMT after 6 weeks (1.45, range 0–3) but not statistically significant. The basal median UPDRS Score was 11.36 (range 10–19) and NMSS was 22.36 (range:14–32). The basal median PAC-QOL score was 102.55 (range 93–108) and the Wexner constipation score was 11.63 (range 5–12). All scores were significantly higher compared to 6 weeks after FMT (Tables 1 and 2).

**Table 2:** Outcome measures in the participants

	Before FMTa	6 weeks After FMT	12 weeks After FMT	p-value
BMI (mm/kg^2^)	22.01 ± 1.73	23.52 ± 1.65	24.65 ± 1.09	
H-Y Grade^**^	2.27 ± 0.75	1.45 ± 0.82	1.09 ± 0.83	*0.0023*
UPDRSII Score^**^	11.36 ± 4.7	6.18 ± 3.6	4.90 ± 3.33	*0.0036*
NMSS^**^	22.36 ± 7.05	12.55 ± 5.54	10.36 ± 4.54	*0.003*
PAC-QOL score^**^	102.55 ± 5.05	51.27 ± 6.71	43.45 ± 5.34	< 0.0001
Wexner constipation score^*^	11.63 ± 3.22	8.16 ± 2.62	6.22 ± 1.03	*0.0231*
HCY (μmol/L)^**^	15.85 ± 2.89	12.74 ± 2.05	11.22 ± 1.85	0.002
Alb (g/L)	38.49 ± 3.92	40.38 ± 4.35	41.62 ± 4.26	
UA (μmol/L)	306.13 ± 75.94	282.09 ± 65.31	274.91 ± 55.73	
OCTT (min)^**^	150.91 ± 12.21	NA	105.45 ± 20.18	< 0.0001

H-Y Grade: Hoehn-Yahr Grade, UPDRS II Score: Unified Parkinson’s Disease Rating Scale II Score. NMSS: non-motor symptom questionnaire, BMI: body mass index (mm/kg^2^), HCY: homocysteine, Alb: albumen, UA: uric acid

^*^ p < 0.05 Before FMTvs 12 weeks After FMT in the same patient

^**^ p < 0.01 Before FMTvs 12 weeks After FMT in the same patient

### Twelve-week assessment

All 11 patients completed the 12 weeks assessment and were able to complete the questionnaires. Median H-Y Grade was 1.09 (SD 0.83; range 0–3; Table 2) at 12 weeks after FMT. Compared to the baseline, 10/11 (90.1%) patients’ scores decreased, with a statistical significance (p = 0.0023; Tables 2 and 3).

**Table 3:**
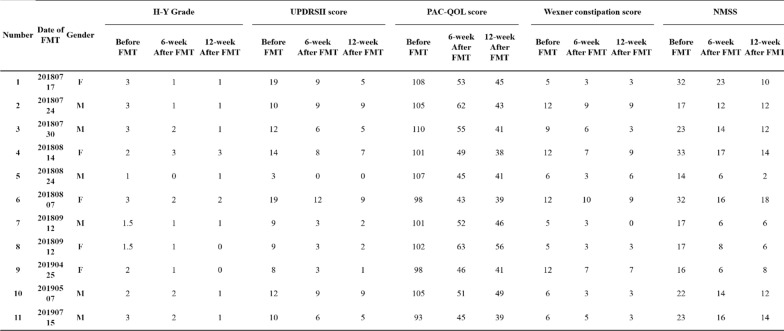
Clinical Assessment Before and After FMT for each patient

H-Y Grade:Hoehn-Yahr Grade, UPDRS II Score: Unified Parkinson’s Disease Rating Scale II Score. NMSS: non-motor symptom questionnaire

The median UPDRS score was 11.36 (SD 4.7; range 8–19; Tables 2 and 3) and NMSS was 22.36 (SD 7.05; range 14–32; Tables 2 and 3) at baseline. The median PAC-QOL score was 102.55 (SD 5.05; range 93–108; Tables 2 and 3) and the Wexner constipation score was 11.63 (SD 3.22; range 5–12; Tables 2 and 3) at baseline. All scores were significantly higher compared to 6 weeks and 12-week after FMT. The median expression of HCY was 11.22 at 12 weeks after FMT compared to the baseline HCY expression (15.85± 2.89), and was statistically different (p = 0.002, Table 2) whereas the Alb and UA differences were not significantly different (p > 0.05, Table 2).

### Efficacy-safety Assessment

An overview of adverse events is shown in Table 4. During treatment, the most common adverse events were mild diarrhea (9.1%), abdominal pain (27.3%), venting (18.2%), flatulence (45.5%), nausea (27.3%), and throat irritation (18.2%) (Table 4). All cases were mild and none resulted in the discontinuation of the treatment. During follow-up, abdominal pain (18.2%) and flatulence (18.2%) was also the most common adverse event. Other adverse events were venting (9.1%) (Table 4) but were self-limiting and not serious.

**Table 4:** Treatment-related adverse events

Adverse event	During treatment	During follow-up
Fever	0	0
Mild Diarrhea	1 (9.1%)	0
Abdominal pain	3 (27.3%)	2 (18.2%)
Venting	2 (18.2%)	1 (9.1%)
Vomiting	0	0
Flatulence	5 (45.5%)	2 (18.2%)
Nausea	3 (27.3%)	0
Throat irritation	2 (18.2%)	0

### The Lactulose H_2_ Breath Test

**Fig. 1:**
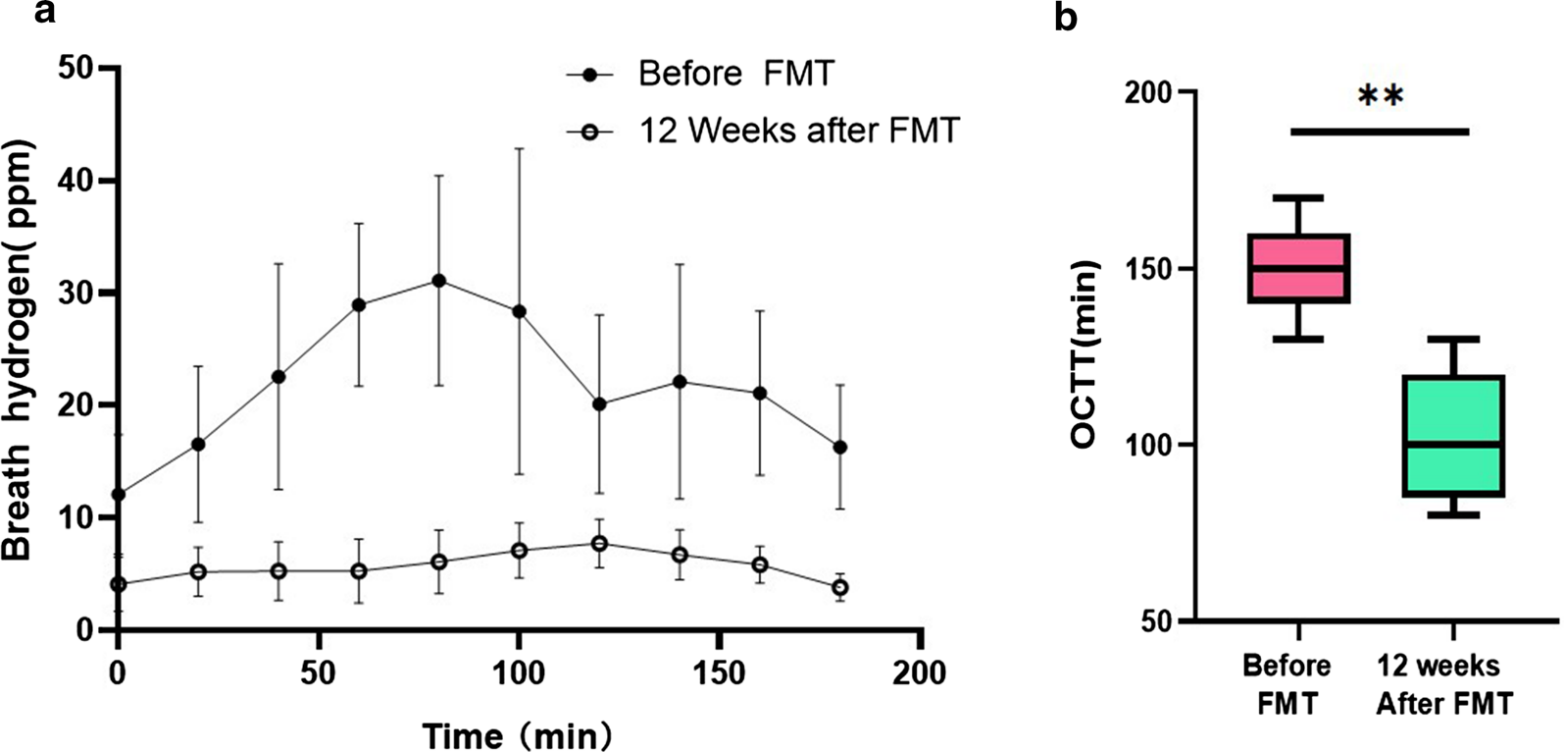
The lactulose H2 breath test in PD patients with FMT. **a** The average breath hydrogen before- and after-FMT. **b** The average orocecal transit time (OCTT) before and after-FMT (**p < 0.01)

The overgrowth of small intestinal bacteria was detected using the lactulose H_2_ breath test before FMT (one week ago) for all PD patients. Twelve weeks after FMT, the overgrowth of small intestinal bacteria appeared corrected (Fig. 1). The average orocecal transit time (OCTT) was 105.45 ± 20.18 min at 12 weeks after FMT. This result was significantly different from the OCTT before FMT (150.91 ± 12.21 min; p < 0.01; Table 2).

### Microbial community structures in before and 12 weeks after FMT in PD patients

**Fig. 2:**
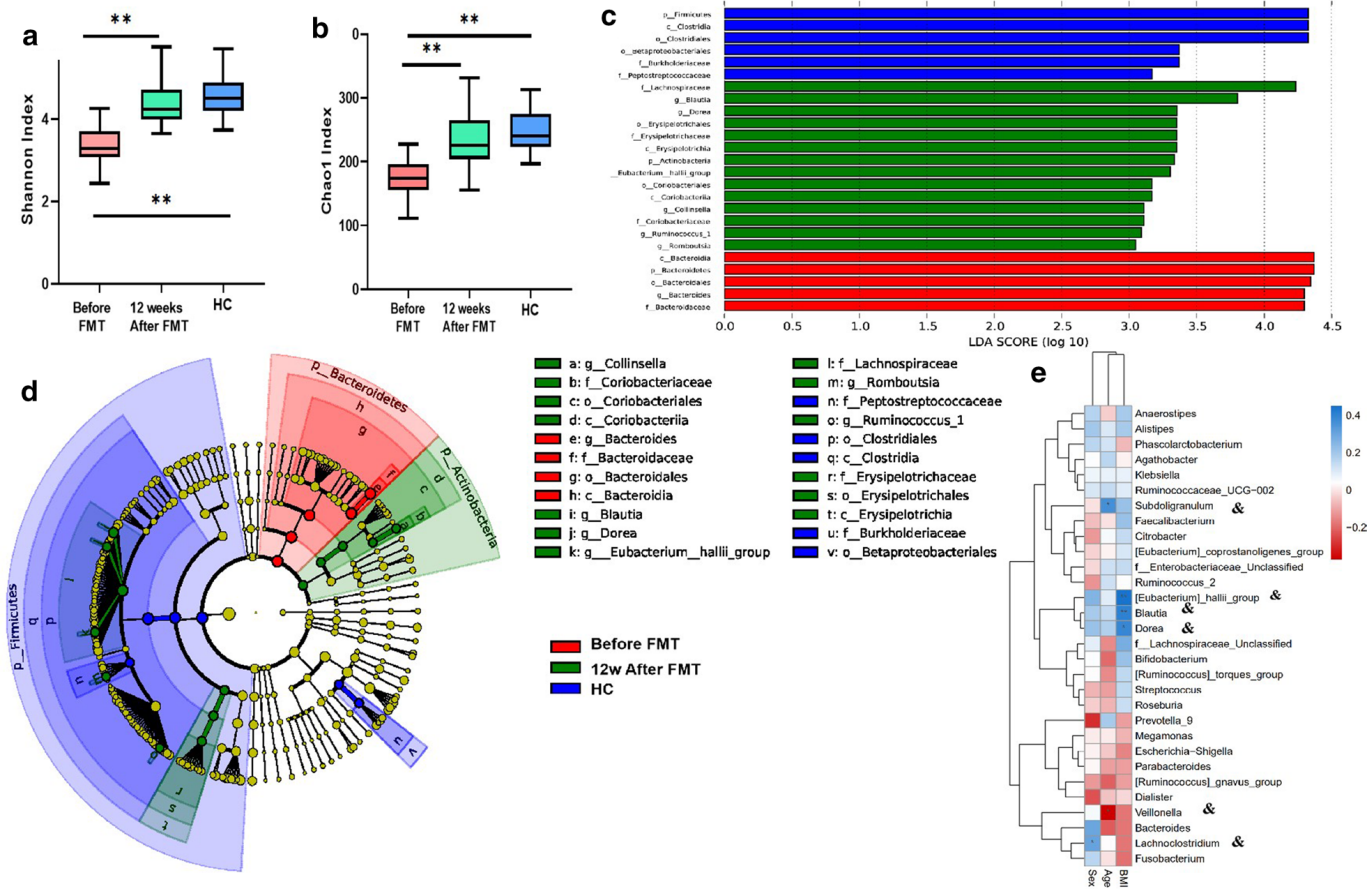
Fecal Microbiota Analysis among healthy controls (marked as HC), PD patients before (marked as before FMT) and 12 weeks after FMT (marked as 12w after FMT). Alpha diversity indices, including **a** diversity (Shannon) and **b** community richness (chao), varied among each group. **c** Histogram of taxonomic profles LDA score of the gut microbiota among healthy controls,PD patients before and 12 weeks after FMT **d** Shotgun sequencing of fecal samples during diagnosis using the LEfSe analysis method with representative relative abundance among each group; and a cladogram representation of taxa enriched in gut microbiota among each group. **e** Associations of gut microbial species with clinical indices: Heat map of the Spearman’s rank correlation coefficient between 3 clinical indices and associated OTUs (Total = 35; control n = 13; before FMT, n = 11,12w after FMT, n = 11); Spearman’s rank correlation. (HC: healthy controls; *p < 0.05; **p < 0.01; ^&^denotes OTUs that significantly differed in abundance in 35 samples (two-tailed Wilcoxon rank-sum test, P < 0.05). BMI, Body Mass Index.)

Changes in intestinal microbiota were analyzed through fecal samples obtained from all of the 11 patients (before FMT and 12 weeks after FMT, respectively) and 13 HCs by the Fecal bacterial 16S rDNA sequencing of the fecal microbiota. We compared the bacterial alpha diversity, including community richness (chao) and diversity (Shannon index), between the PD patients (before and 12 weeks after FMT) and HCs. Significant differences in the diversity indexes were observed, not only between the pre-FMT and 12 weeks after FMT (P < 0.01; Fig. 2a, b) but also between pre-FMT and HCs (P < 0.01; Fig. 2a, b). The species diversity and the pattern of the richness of pre-FMT PD patients were significantly decreased compared to 12 weeks after FMT and HCs (P < 0.05). However, the richness and the diversity indices in 12 weeks after FMT was not significantly different from HCs.

Subsequently, linear discriminant analysis efect size (LEfSe) was used to identify diferential microorganism communities between groups. The LDA score (Fig. 2c) and cladogram (Fig. 2d) showed that the phyla *Bacteroidetes*, genus *Bacteroides*, order_*Bacteroidales*, class *Bacteroidia*, family Bacteroidacea were the dominant bacteria in the PD patients before FMT. However, at the family level,*Coriobacteriaceae*, *Erysipelotrichaceae* and *Lachnospiraceae*, which belonging to the Phyla *Actinobacteria* and *Firmicutes* respectively,were the dominant bacteria in the fecal microbiota of PD patients at 12 weeks after FMT. While at the genus level, *Collinsella*, *Eubacterium__hallii_group*, *Ruminococcus_1*,*Dorea*, *Blautia*, *Romboutsia* were the dominant bacteria in the fecal microbiota of PD patients at 12 weeks after FMT.

To identify correlations between clinical parameters and changes in the gut microbiome in obesity, we performed the heat map of the Spearman’s rank correlation coefficient between.

3 clinical indices and associated OTUs. We found that these OTUs gut microbiome were associated with clinical parameters, including age, year and BMI, by Spearman’s correlation (Spearman’s correlation value < -− 0.3 or > 0.3, adjusted p < 0.05).* [Eubacterium]_hallii_group* (p < 0.01, Fig. 2e),*Blautia* (p < 0.01,  Fig. 2e) and *Dorea* (p < 0.05, Fig. 2e) were positively correlated related to BMI.OTUs for *Subdoligranulum* was positively correlated with age (p < 0.05,Fig. 2e), and *Veillonella* was negatively correlated with age (p < 0.05, Fig. 2e).OTU for *Lachnoclostridium* was positively with sex (p < 0.05, Fig. 2e).

### Overall taxonomic analysis of fecal microbiota

**Fig. 3:**
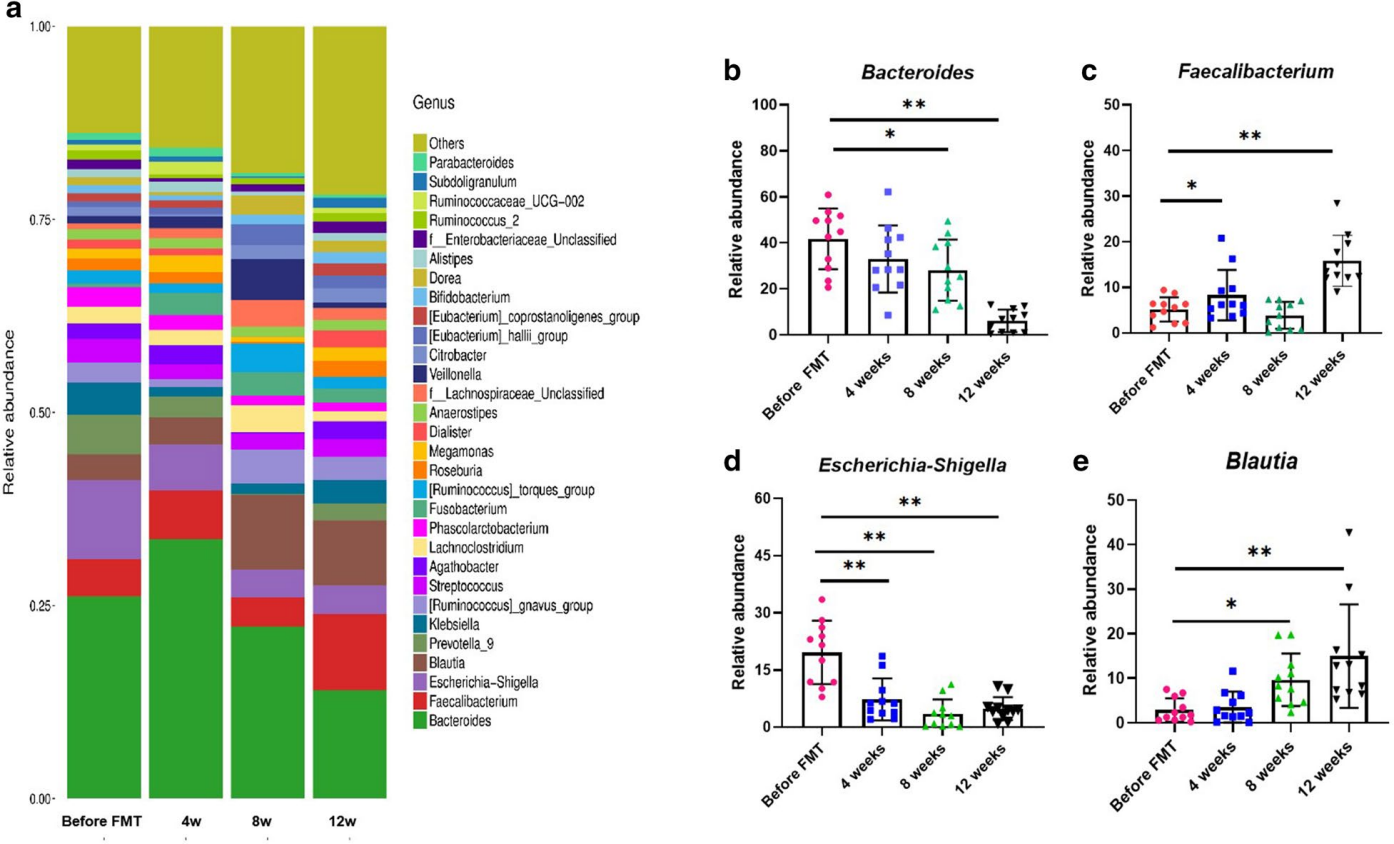
Changes in taxonomic composition distribution in different samples of genus-level before- and after-FMT. **a** Distribution of top 30 species at the genus level; **b** *Bacteroides*. **c** *Faecalibacterium.* **d** *Escherichia–Shigella.* **e** *Blautia*. (**p < 0.01, vs Before FMT, *p < 0.05 vs Before FMT)

Upon further analysis of the alterations at the genus level between before- and after-FMT, we observed that the dominant genus was the *Bacteroides*, belonging to the *Bacteroidetes* in PD patients before FMT (Fig. 3a). However, the *Bacteroides* gradually and significantly decreased in before-FMT PD patients compared to that in the 8-week and 12-week after-FMT groups (before FMT vs 8 weeks after FMT p < 0.05; before FMT vs 12 weeks after FMT p < 0.01; Fig. 3b), but without statistical significance when compared to the 4 week after-FMT group (p = 0.518; Fig. 3b). *Escherichia − Shigella* also decreased gradually in before-FMT PD patients compared to the other three after-FMT groups (before FMT vs 4 weeks after FMT p < 0.05; before FMT vs 8 weeks after FMT p < 0.01; before FMT vs 12 weeks after FMT p < 0.01; Fig. 3d).

Furthermore, *Faecalibacterium* increased at 4 and 12 weeks after FMT (before FMT vs 4 weeks after FMT p = 0.026; before FMT vs 12 weeks after FMT p < 0.01 Fig. 3b), whereas it appeared to decrease compared to the 8 week after-FMT group, without statistical significance (p = 0.433; Fig. 3c). Similarly, *Blautia* increased at 8 weeks and 12 weeks after FMT (before FMT vs 8 weeks after FMT p < 0.01; before FMT vs 12 weeks after FMT p < 0.01; Fig. 3e), whereas it increased nominally in the 4-week after-FMT group but without any statistical significance (p = 0.372; Fig. 3e).

## Discussion

Parkinson’s disease is a neurodegenerative disorder and is always accompanied by constipation. The intestinal immune system plays an essential role in maintaining intestinal immune homeostasis, mediating immune responses, and regulating inflammation [15].

Accumulating evidence suggests a direct impact of the gut microbiota and microbial metabolites on PD pathogenesis [16]. Both Meng-Fei Sun et al. [17]. and Sampson et al. [18] demonstrated that gut microbiota not only affects the motor symptoms but also brain function in a PD model.

The concept of the “gut microbiota-brain axis” is well established and its dysregulation may lead to neurological diseases such as PD [19]. Microbiota seems to play an important role in the occurrence and development of neurological diseases and evidence indicates a potential bidirectional interaction between the gut microbiota and PD [20]. Studies show that the gut microbial composition of PD patients is different from HCs [21]. Changes in gut microbiota have also been observed in PD animal models with motor deficits and neuroinflammation [22].

Gastrointestinal dysfunction is one of the most common non-motor symptoms in PD, especially constipation [23]. Studies suggest that neurotoxic substances with prion-like properties may be improperly folded.α-Syn a typical pathogenic agent for PD, is transported from the gastrointestinal tract to the central nervous system during the early stages of PD [24].

Sampson et al. [18] showed that gut dysbiosis leads to an altered ratio of short-chain fatty acids (SCFA). SCFA modulates the activity of the ENS and increases gastrointestinal motility [25], especially butyrate, and alters the microglial signaling in the brain leading to disease development and the appearance of PD-associated motor symptoms [26]. Hence, the altered concentrations of SCFA might contribute to the gastrointestinal dysmotility in PD. In recent years, an increasing number of studies have shown that the levels of certain gut microbiota differ between PD patients and HCs [27].

FMT is a well-established treatment for the reconstruction of gut microbiota [28] and has been proposed as a therapeutic option for functional gastrointestinal disease [8]. It can repair the disruption of the normal microbial communities for the efficient treatment of metabolic disorders [29]. Dae-Wook Kang et al. [30] found that microbiota transfer therapy led to significant improvements in both GI- and ASD-related symptoms, and the improvements were sustained for at least 8 weeks after the treatment. As similar to research by Sampson [18],which found that fecal microbiota transplantation from PD patients, compared to microbiota from HCs, exacerbated the α-Syn mediated motor dysfunction in ASO mice, demonstrating that gut microbiota can influence brain function in PD.

At our center, FMT treatment has been used for PD patients with gastrointestinal dysfunction since 2018. In 2019, Hongli et al. [9] reported successful FMT treatment in a PD patient with constipation, which encouraged us to summarize and analyses the FMT-treated patients from the last two years. In our study, remission of the constipation symptoms was observed in all patients, which might be related to increased microbial abundance. Here we report alterations in the gut microbiota composition that reproduce some of these previously reported findings among PD patients, before and after FMT, and HCs using the 16S rDNA sequencing analysis. We observed a decrease in the community abundance of fecal microbiota and the microbial diversity was lower in before-FMT PD patients compared to the after-FMT and HCs; all differences were statistically significant.

The abundance of *Blautia* and *Lachnospiraceae* (Phylum: *Firmicutes*) was significantly increased in the after-FMT groups comparing to the before-FMT PD patients. We observed a significantly increased abundance of *Bacteroides* (phylum: *Bacteroidetes*) and a significantly reduced abundance of *Faecalibacterium* (phylum: *Firmicutes*) among the PD patients, before and after FMT, compared to the HCs. *Faecalibacterium prausnitzii* is the representative species of *Faecalibacterium* that produce butyrate and is a beneficial gut bacterium with anti-inflammatory properties; its levels are reduced in PD patients [31]. The significant reduction in the abundance of *Faecalibacterium* in PD patients before FMT in our study is consistent with the previous study [32]. Besides, Keshavarzian et al. found Bacteroidetes to be positively correlated with the PD duration [15]. Our results were similar to theirs. However, we observed no correlation between the PD duration and the abundance of *Bacteroidetes* or *Firmicutes*. Further studies are required due to the limited number of cases.

Similar to the study by Keshavarzian, we also observed an increased abundance in *the Enterobacteriaceae (phylum: Proteobacteria)* in PD patients before FMT. Scheperjans et al. [21] observed that the relative abundance of *Enterobacteriaceae* in PD patients was positively correlated with postural instability and gait difficulty. And another recent study reported an increase of Enterobacteriaceae family in PD patients with akinetic rigid motor phenotype compare to PD patients with tremor dominant phenotype. In our research [33], the abundance of *Escherichia–Shigella* (Family: *Enterobacteriaceae*) also gradually decreased in the three after-FMT groups compared to the before-FMT PD patients. Similarly, we observed that postural instability and gait difficulty improved and that the H-Y grade, UPDRS, and NMSS of PD patients decreased significantly after FMT. Therefore, a correlation may exist between the changes of the abundance of Escherichia–Shigella and the clinical symptoms, and that FMT may have a positive impact on the relief of clinical symptoms in PD patients.

In addition, we also observed a decreased abundance of *Blautia* (affiliated with *Firmicutes* phylum), butyric acid-producing bacteria, in PD patients before FMT. According to the analysis of data by heat map of the Spearman’s rank correlation and taxonomic composition distribution before and after-FMT, we speculate the decreased abundance of *Blautia* might relate to the FMT or BMI. Further studies are needed to confirm the findings and to uncover the possible mechanisms behind the association.

Recent studies shown that bacteria can producing SCFA, which was reduced in PD patients. SCFA such as butyrate modulates the activity of the ENS and thereby increase the gastrointestinal motility [32]. Hence, changing the concentrations of SCFA might contribute to gastrointestinal dysmotility in PD. In our study, the remission constipation symptoms were observed in all PD patients after FMT and the benefits continued for at least 12 weeks. Thus, it appears that FMT treatment might lead to significant improvements in GI symptoms in PD patients. Further studies are needed to further clarify this.

Gut microbiota and their metabolic products are potential candidates that could initiate a process, eventually leading to Lewy body formation in the ENS [34]. As the progression of PD course, dysfunction of ENS (dysautonomia) increases which leads to slowing of GI motility, which in turn predisposes the PD patients to small intestinal bacterial overgrowth (SIBO) [35].

The role of SIBO has been studied by Fasano et al. [36], who showed that the prevalence of SIBO is higher in PD patients than the HCs. In our study, SIBO correction with the FMT (after 12 weeks) resulted in improvement of GI symptoms along with improvement in the motor fluctuations (p < 0.05). This suggests that FMT may be able to reverse the phage-mediated dysbiosis of the PD gut, although further studies are required to confirm this assertion.

FMT is considered to be a safe treatment. Previous studies show that almost all of the common adverse events, such as venting, abdominal pain, bloating, and diarrhea, disappeared after 12 weeks [36]. In our study, we did not observe any adverse events, such as fever, abdominal pain, hypoxia, paroxysmal atrial fibrillation (PAF), transplant-related lower gastrointestinal bleeding, cholestasis, liver damage. Xiaofei Qi et al. [37] reported that a patient treated with FMT for sterile refractory intestinal acute graft-versus-host disease experienced thrombocytopenia after FMT. We did not observe this in any of our 11 patients.

Based on our results, FMT is a good choice for PD treatment with gastrointestinal symptoms; however, its effectiveness and safety requires further evaluation. In order to evaluate the effectiveness and safety of FMT in the treatment of PD accurately, a larger sample study is required in the future.

This tentative study may open a new avenue to study the mechanism of the microbe-gut-brain axis and the biological treatment of PD. The current use of FMT to treat PD is beginning and it has inspired us to further our understanding of FMT in treating neurological diseases.

In conclusion, our patients demonstrated efficient reliving of PD constipation by FMT and its positive impact on clinical characteristics. The changes before and after FMT suggest the potential for a more targeted and specific FMT therapy in PD. FMT can be used for the treatment of PD with gastrointestinal symptoms, but its effectiveness and safety requires further evaluation.
